# Business Houses and the Hospitals

**Published:** 1920-09-11

**Authors:** 


### BUSINESS HOUSES AND THE HOSPITALS.

Our repeated assertion that collections from the business houses in London can be organised much more methodically than at present is borne out by the speech which Mr. R. Holland-Martin made at the recent meeting of the Council of the Metropolitan Hospital Sunday Fund. We make no apology for repeating the following passage from his speech: "The Lord Mayor had given the Fund help in a special way. He made a 'drive' amongst business houses, and at Lloyd's, the Stock Exchange, and the Baltic. It was due to him and the energy of its chairman that Lloyd's had contributed no less than £5,045. The Baltic and the Stock Exchange had also opened special collections, which had been generously subscribed to, and large increases were recorded. A great deal of help had also come from business houses, several of which gave large amounts. He was confident that good results would be obtained if large business houses would open collections in their offices."

The problem is to make these collections the rule rather than the exception, and the question arises whether the attempt should be made by separate hospitals or by the Saturday or Sunday Funds, the latter of which, as we have seen, has already begun to move in this direction. At present the matter does not seem to have passed beyond the stage of agreeing to a collection, but since the Fund to which it is sent is an annual Fund, the collections presumably will be maintained each year. From this it is but a step to the establishment of a hospital fund for each house of business, and the difference between such a fund and a collection is the difference between continuous and sporadic generosity. In the case of the business houses, anyway, the total is likely to be much larger if small contributions are collected regularly throughout the year in time to be paid over at a fixed date, and it should not be impossible to adopt somewhat the same plan at Lloyd's or the Stock Exchange. In the case of the latter corporations the committee in charge of the collections might set out to organise annual subscriptions from the members, and if the list were given a good start an enthusiastic committee could probably contrive that every member's name was eventually placed upon it. In regard to the business houses, those which have already started collections should know better than anyone how to persuade other firms to follow suit. The success of the Sunday Fund this year is an object-lesson, because, though the churches have "done a great deal better," it seems clear that the high total would not have been reached had it not been for the collections both from working men and from the business houses. The success shows what can be done, and also how much has not been done in the past. There can be no reason why the small regular contribution which plays so large a part in the provinces should not be developed much more thoroughly in London. The success of the Sunday Fund collection should teach us how this has been brought about.

